# An encoded N-terminal extension results in low levels of heterologous protein production in *Escherichia coli*

**DOI:** 10.1186/1475-2859-4-22

**Published:** 2005-07-21

**Authors:** Samantha S Orchard, Heidi Goodrich-Blair

**Affiliations:** 1 Department of Bacteriology, University of Wisconsin, Madison, WI 53706, USA; 2 Department of Biology, San Diego State University, San Diego, CA 92182 USA

## Abstract

**Background:**

The *tdk *gene (encoding deoxythymidine kinase) of the gamma-proteobacterium *Xenorhabdus nematophila *has two potential translation start sites. The promoter-distal start site was predicted to be functional based on amino acid sequence alignment with closely related Tdk proteins. However, to experimentally determine if either of the two possible start codons allows production of a functional Tdk, we expressed the "long-form" (using the promoter-proximal start codon) and "short-form" (using the promoter-distal start codon) *X. nematophila tdk *genes from the T7 promoter of the pET-28a(+) vector. We assessed Tdk production and activity using a functional assay in an *Escherichia coli tdk *mutant, which, since it lacks functional Tdk, is able to grow in 5-fluorodeoxyuridine (FUdR)-containing medium.

**Results:**

Short-form Tdk complemented the *E. coli tdk *mutant strain, resulting in FUdR sensitivity of the strain. However, the *E. coli tdk *mutant expressing the long form of *tdk *remained FUdR resistant, indicating it did not have a functional deoxythymidine kinase enzyme. We report that long-form Tdk is at least 13-fold less abundant than short-form Tdk, the limited protein produced was as stable as short-form Tdk and the long-form transcript was 1.7-fold less abundant than short-form transcript. Additionally, we report that the long-form extension was sufficient to decrease heterologous production of a different *X. nematophila *protein, NilC.

**Conclusion:**

We conclude that the difference in the FUdR growth phenotype between the *E. coli tdk *mutant carrying the long-or short-form *X. nematophila tdk *is due to a difference in Tdk levels. The lower long-form protein level does not result from protein instability, but instead from reduced transcript levels possibly combined with reduced translation efficiency. Because the observed effect of the encoded N-terminal extension is not specific to Tdk production and can be overcome with induction of gene expression, these results may have particular relevance to researchers attempting to limit production of toxic proteins under non-inducing conditions.

## Background

Proteins from one organism are often expressed in a different species for the purpose of protein purification or complementation studies. When such efforts fail due to non-production of the protein, the underlying cause of failure is often unclear [1]. Protein overproduction is known to induce a heat shock-like response, which results in increased proteolysis in the cell and therefore possible degradation of the desired protein [2]. Other factors such as degradation of the RNA transcript, efficiency of translation and toxic nature of the desired protein may also influence the level of protein production. Thus, common *Escherichia coli *strains used for protein overproduction include protease mutants (*e.g*. BL21; *lon*), RNase mutants (*e.g*. BL21 Star™; RNaseE mutant strain; Invitrogen, Carlsbad, CA) and those that provide tRNA synthetases corresponding to infrequently used codons (*e.g*. Rosetta™; Novagen, Madison, WI) to increase translational efficiency. To reduce production of potentially toxic proteins at inappropriate points in the growth of the host strain, heterologous genes are often fused to engineered promoters that limit gene expression under non-inducing conditions. However, all promoters have a degree of "leakiness" and allow some protein production even under non-inducing conditions.

The predicted *Xenorhabdus nematophila *Tdk protein is 70% identical to *E. coli *Tdk and has been shown to have deoxythymidine kinase activity [3], converting salvaged deoxythymidine to deoxythymidine monophosphate [4]. A translational start site was predicted for *X. nematophila tdk *based on alignment with *tdk *sequences from other organisms. However, *X. nematophila tdk *has an additional potential start codon 12 bp 5' from the predicted start site. As part of an effort to establish which start codon is used for native *X. nematophila *Tdk synthesis, the two forms were expressed from a heterologous promoter in *E. coli*. These studies revealed that, in contrast to short-form, long-form Tdk is not expressed. Furthermore, the additional four codons present in long-form Tdk are sufficient to decrease production of another unrelated protein, NilC.

## Results and discussion

### Long-form Tdk does not confer FUdR sensitivity to an *E. coli tdk* strain

**Figure 1:**
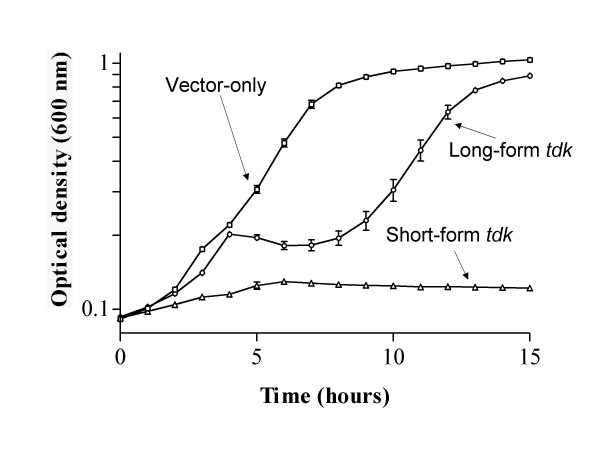
Growth of *E. coli *KY895 carrying plasmids pTara and pET-28a(+) (squares), pETSTdk (triangles) or pETLTdk (circles) in FUdR-containing medium. Each data point represents the average optical density reading for six independent cultures for each strain. Error bars represent standard error of the mean.

To determine if both short-and long-form (with four extra amino acids) Tdk protein are functional, they were expressed in an *E. coli tdk *mutant strain (KY895) and their activity assessed in an FUdR growth assay. In an environment lacking in salvageable deoxythymidine and containing FUdR, cells with active Tdk are starved for thymidylate due to inhibition by Tdk-phosphorylated FUdR (FdUMP) of the endogenous synthesis pathway and thus cannot grow (Tdk^+ ^= FUdR^s^). pETSTdk, the plasmid used to express short-form *tdk*, complemented the *tdk *defect in KY895, as demonstrated by the resulting FUdR sensitivity of the strain (Fig. 1; triangles). The FUdR sensitivity of the strain carrying plasmid pETSTdk was similar to that of a strain carrying wild-type *E. coli tdk *(data not shown). Thus, short-form Tdk appears to be expressed and active in this system. In contrast, KY895 carrying plasmid pETLTdk, designed to express long-form *tdk*, had an extended lag phase during growth in FUdR punctuated by a reproducible brief increase then decrease in optical density (Fig. 1; circles). This strain ultimately exhibited the same optical density (FUdR resistance) as the strain carrying the control vectors (Fig. 1; squares) and grew similarly to KY895 carrying plasmid pETSTdk or pET28a(+) in Luria-Bertani broth (LB [11]; data not shown). Two possible explanations for this lack of *tdk *complementation in FUdR-containing medium are that long-form Tdk was not adequately produced in this system or that the long-form protein was not active. Further experiments were aimed at distinguishing between these two possibilities.

### Long-form Tdk is not detected by immunoblot analysis

**Figure 2:**
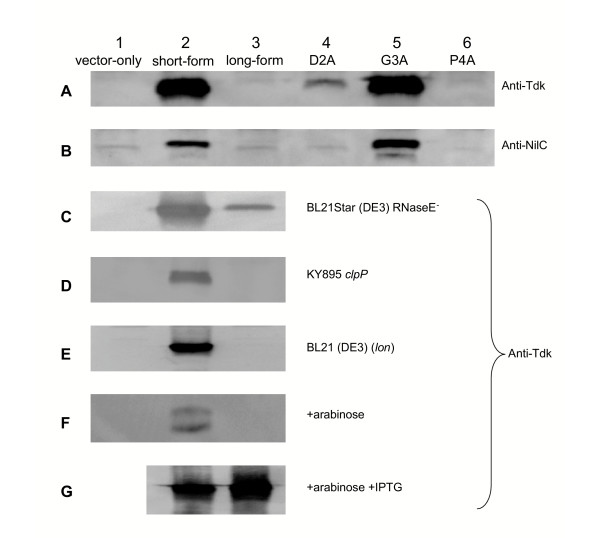
Immunoblot analysis of protein production using anti-Tdk serum (panels A and C-G) or anti-NilC serum (panel B). *E. coli *KY895 (panels A, B, F, G), BL21 Star™ (DE3) (panel C), KY895 *clpP *(panel D), or BL21 (DE3) (panel E) carrying plasmids pTara and pET-28a(+) (vector-only; lane 1), pETSTdk or pETSNilC (short-form; lane 2), pETLTdk or pETLNilC (long-form; lane 3), pETMAGPTdk or pETMAGPNilC (D2A change; lane 4), pETMDAPTdk or pETMDAPNilC (G3A change; lane 5) or pETMDGATdk or pETMDGANilC (P4A change; lane 6) was grown overnight in LB/kan/cam and separated cellular proteins were subjected to immunoblot analysis with the indicated antiserum. Arabinose (0.2%) was added to the culture medium for the strains in panels F and G, and IPTG (0.2 mM) was added to the culture medium for the strains in panel G. Similar volumes of cultures were used in each lane but cultures were not normalized for optical density or overall protein concentrations.

Immunoblot analysis with polyclonal anti-Tdk serum of overnight cultures of *E. coli *carrying plasmids pTara (encoding arabinose-inducible T7 polymerase) and pETSTdk or pETLTdk failed to reveal long-form Tdk even though short-form Tdk was readily detected (Fig. 2A). These results indicate that the inability of pETLTdk to complement the *tdk *mutation in *E. coli *for growth in FUdR (above) was likely due to non-production of long-form Tdk and not necessarily lack of activity in long-form Tdk. Since the only difference between the long-and short-form proteins is the set of additional amino acid residues (MDGP) at the N-terminus, we concluded that these residues or the DNA or mRNA encoding them minimize *tdk *expression.

### Long-form NilC-trunc is not detected by immunoblot analysis

To determine if the 5' extension of long-form *tdk *would reduce production of a different protein, the 12-nt extension was added to an *X. nematophila *gene, *nilC*, encoding an outer membrane-associated protein [5] and for which there were readily available antibodies. To create a cytoplasmic version of NilC, the DNA encoding the 21 amino acid long NilC N-terminal signal sequence was removed creating NilC-trunc (short form), to which the MDGP extension was added (long form). Long-form NilC-trunc was not detected by immunoblot analysis with polyclonal anti-NilC serum while short-form NilC-trunc was (Fig. 2B). Thus, the MDGP-encoding extension is sufficient to reduce production of at least two unrelated proteins.

### Long-form *tdk *transcript is less abundant than short-form transcript but shares the same 5' end

**Figure 3:**
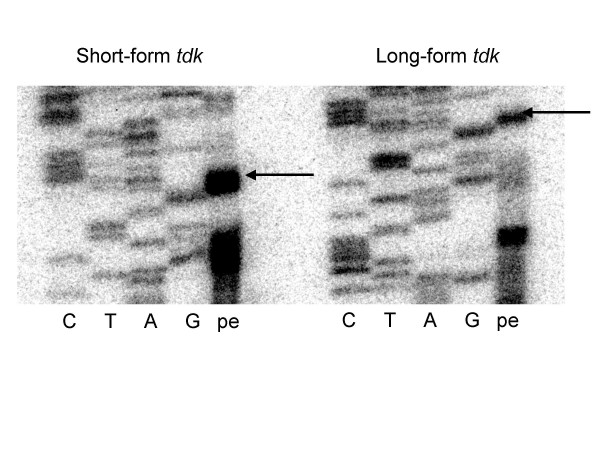
Primer extension analysis of short-and long-form *X. nematophila tdk *transcripts in *E. coli*. Total RNA was isolated from *E. coli *KY895 carrying plasmids pTara and pETSTdk or pETLTdk grown overnight to OD_600 _= 2.0. Primer tdkprext2 was end-labeled with ^32^P and used to produce a sequencing ladder from plasmids pETSTdk (left side; lanes C, T, A, G) and pETLTdk (right side; lanes C, T, A, G). A primer extension product was produced with end-labeled tdkprext2, total RNA template (left side: KY895 carrying pETSTdk; right side: KY895 carrying pETLTdk) and AMV-RT enzyme at 37°C (lanes "pe"). Arrows indicate the expected transcriptional start site from the T7 promoter of pET-28a(+).

Primer extension analysis was used to determine if the MDGP-encoding sequence affects the transcription (either level or start site) of *tdk *from pET-28a(+) or processing of the *tdk *transcript. Primer extension analysis indicated that long-form *tdk *transcript was made and at least some portion maintained its 5' end (Fig. 3). Relative transcript levels were quantified by real-time PCR with primers specific for a central portion of the *tdk *gene. For growth in LB broth to OD_600_~0.85, the long-form transcript was 1.7-fold less abundant that the short-form transcript. This lower level of long-form *tdk *transcript could be due to higher levels of long-form *tdk *mRNA degradation, reduced transcription of long-versus short-form *tdk *or to differences in plasmid copy number, possibly a result of the presence of long-form *tdk *or long-form Tdk production. To determine if this lower transcript abundance is due to increased susceptibility to degradation, protein production was monitored in an *E. coli *RNaseE mutant. RNaseE is one of the main *E. coli *mRNA-degrading enzymes [6] and we hypothesized that it differentially degrades long-versus short-form *tdk *transcript. In the RNaseE mutant, long-form Tdk was detected at low levels by immunoblot analysis (Fig. 2C). Thus in the absence of RNaseE, long-form *tdk *transcript was sufficiently produced and stable to act as a template for protein synthesis, indicating that RNaseE degrades long-form *tdk *transcript. However, even in the absence of RNaseE, long-form Tdk protein levels were ~4.7-fold lower than those of short-form Tdk produced under the same conditions. Therefore, RNaseE-mediated degradation of long-form *tdk *transcript is not the only factor contributing to the low levels of long-form Tdk protein production relative to short-form Tdk.

### Long-form Tdk protein is stable

**Figure 4:**
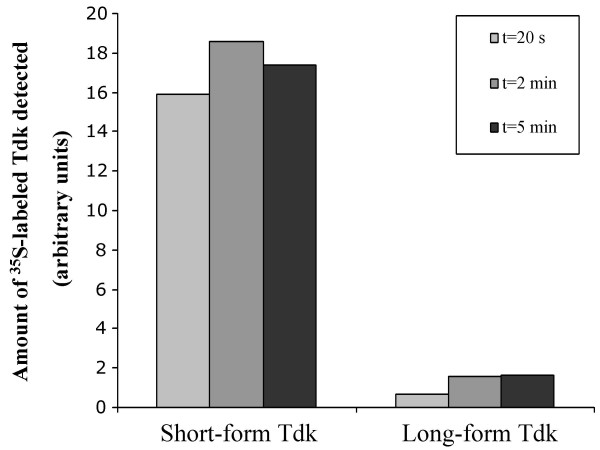
Quantification of Tdk protein levels and stability. Levels of protein were determined by ^35^S-methionine labeling of whole cells and subsequent immunoprecipitation with polyclonal anti-Tdk antiserum. *E. coli *KY895 carrying plasmids pTara and pETSTdk (Short-form Tdk) or pETLTdk (Long-form Tdk) was labeled with ^35^S-methionine for 3 min followed by a chase with excess unlabeled methionine. Samples were removed for processing at 20 s, 2 min and 5 min following the chase and proteins reactive with anti-Tdk serum were immunoprecipitated and separated as described in Methods. Following autoradiography, intensity of the bands corresponding to labeled Tdk were determined and background corrected against a pET-28a(+) vector-only control. The calculated band intensities are expressed in arbitrary units.

The stabilities of short-and long-form Tdk proteins were measured to determine if differential susceptibility to post-translational proteolysis is the cause of the low protein levels of long-versus short-form Tdk. Measurement of long-form Tdk stability was made possible by inducing *tdk *expression by addition of arabinose to the culture medium (arabinose induces expression of pTara-encoded T7 RNA polymerase, which is responsible for transcription from the pET-28a(+) promoter controlling *tdk *expression). Pulse-chase labeling of cells and immunoprecipitation experiments revealed that long-form Tdk protein is present at a low level with such induction (Fig. 4), although this level was still 13-fold lower on average than that of short-form Tdk and was insufficient for detection by immunoblotting (Fig. 2F). Over the 5 min time course of the experiment, long-form Tdk was at least as stable as short-form Tdk (Fig. 4), indicating that the difference in protein levels between these two forms is not due to differential proteolysis. Further support for this hypothesis comes from the fact that long-form Tdk was not detected in *E. coli *KY895 *clpP *and *E. coli *BL21(DE3) (*lon*) protease mutant strains (Fig. 2D–E).

### Long-form Tdk is expressed in the presence of IPTG and arabinose

Arabinose was used to induce T7 RNA polymerase for the ^35^S-methionine labeling study, resulting in detectable long-form Tdk protein levels by autoradiography (Fig. 4) but not immunoblot analysis (Fig. 2F). Neither arabinose nor IPTG (de-repression of the pET-28a(+) T7 promoter) were used for the other protein production studies reported herein. When both IPTG and arabinose were added to cultures of KY895 carrying pTara and pETLTdk, long-form Tdk was detected by immunoblot analysis at levels similar to short-form Tdk in comparably induced cultures (Fig. 2G). Thus, increased transcription of long-form *tdk *is sufficient to promote high long-form protein levels. These data support the hypothesis that the low level of protein expressed from genes with the MDGP-encoding extension results at least in part from a reduction in mRNA levels. These results further indicate that, in non-inducing conditions in cases when overproduction of a toxic protein is desired, the encoded N-terminal extension described here might be beneficial in reducing "leaky" protein production.

### Changing the nucleotide and/or amino acid identity of the long-form extension results in variable protein production

To determine the relative importance of each codon in the extension region of long-form *tdk *and *nilC*-trunc to the resulting observed protein levels, we systematically changed the aspartic acid, glycine and proline codons to alanine codons and monitored the resulting effects on protein production. The levels of the altered long-form proteins were assessed by immunoblot analysis following normalization of samples by optical density (Table 2; see also Fig. 2A–B). The only change that caused an increase in the level of both long-form Tdk and NilC-trunc protein production was a glycine (GGG) to alanine (GCG) codon change (Fig. 2A and 2B). An aspartic acid (GAC) to alanine codon (GCC) change did not alter the level of production of long-form NilC-trunc but did allow detection of long-form Tdk (Fig. 2A). Additional changes were made in the long-form extension region to assess the importance of the position and order of the amino acid residues, particularly glycine (Table 2). All extensions that result in low levels of protein production (similar to that of MDGP) include a glycine codon in the third or fourth position, even when the initiating methionine residue is predicted to be cleaved from synthesized proteins (see Table 2 for a list of extensions where the N-terminal *f*-Met residue is expected to be cleaved by PepM following protein synthesis [7]). Thus, the position of the glycine residue or codon nucleotides appears to impact long-form protein production.

**Table 2:** *X. nematophila tdk *plasmid design and resulting Tdk protein levels

**Oligonucleotide name**	**Oligonucleotide sequence (5'->3') ^a^**	**Resulting plasmid name**	**N-terminus of engineered protein^b^**	**Relative amount of protein expressed (% of short-form) ^c^**
NcoITdkshort	CCATGGCTCAGCTTTATTTTTAT	pETSTdk	*M*AQLY...	100
NcoITdklong	CCATGGACGGGCCAATGGCTCAG	pETLTdk	MDGPMAQLY...	0
NcoIMDGATdk	CCATGGACGGGGCAATGGCTCAG	pETMDGATdk	MDGAMAQLY...	0
NcoIMDPGTdk	CCATGGACCCAGGGATGGCTCAGCTTTATTTTTATTATTCTGC	pETMDPGTdk	MDPGMAQLY...	0
NcoIMDG(GGU)PTdk	CCATGGACGGTCCAATGGCTCAG	pETMDG(GGU)PTdk	MDGPMAQLY...	0
NcoIMAAGTdk	CCATGGCCGCCGGGATGGCTCAGCTTTATTTTTATTATTCTG	pETMAAGTdk	*M*AAGMAQLY...	0
NcoIMDAGTdk	CCATGGACGCCGGGATGGCTCAGCTTTATTTTTATTATTCTG	pETMDAGTdk	MDAGMAQLY...	0.2
NcoIMAGPTdk	CCATGGCCGGGCCAATGGCTCAG	pETMAGPTdk	*M*AGPMAQLY...	1
NcoIMGPDTdk	CCATGGGGCCAGACATGGCTCAGCTTTATTTTTATTATTCTGC	pETMGPDTdk	*M*GPDMAQLY...	13
NcoIMAAAGTdk	CCATGGCCGCCGCCGGGATGGCTCAGCTTTATTTTTATTATTCTG	pETMAAAGTdk	*M*AAAGMAQLY...	15
NcoIMAPGDTdk	CCATGGCCCCAGGGGACATGGCTCAGCTTTATTTTTATTATTCTG	pETMAPGDTdk	*M*APGDMAQLY...	47
NcoIMGDPTdk	CCATGGGGGACCCAATGGCTCAGCTTTATTTTTATTATTCTGC	pETMGDPTdk	*M*GDPMAQLY...	77
NcoIMGAATdk	CCATGGGGGCCGCCATGGCTCAGCTTTATTTTTATTATTCTG	pETMGAATdk	*M*GAAMAQLY...	80
NcoIMAGATdk	CCATGGCCGGGGCCATGGCTCAGCTTTATTTTTATTATTCTG	pETMAGATdk	*M*AGAMAQLY...	88
NcoIMDAPTdk	CCATGGACGCGCCAATGGCTCAG	pETMDAPTdk	MDAPMAQLY...	93
NcoIMAPDGTdk	CCATGGCCCCAGACGGGATGGCTCAGCTTTATTTTTATTATTCTG	pETMAPDGTdk	*M*APDGMAQLY...	103

^a^Underlined nucleotides indicate engineered restriction sites used in cloning ^b^An italicized *M *indicates an N-terminal *f*-Met residue that, based on the identity of the second amino acid residue, is predicted to be posttranslationally removed by PepM ^c^Amount of Tdk protein detected by immunoblot analysis of *E. coli *KY895 carrying pTara and the pET-28a(+)-derived plasmid listed and expressed relative to the level of detected short-form Tdk and background corrected against the pET-28a(+) vector-only level.

## Conclusion

A version of *X. nematophila *Tdk protein with four extra amino acids (long-form Tdk) was not detected by immunoblot analysis of cells carrying plasmid pETLTdk even though the cells could express Tdk lacking these extra amino acids (short-form Tdk encoded on pETSTdk) under the same non-inducing growth conditions. Even with arabinose induction of transcription, long-form Tdk protein was present at a level ~13-fold lower than the short-form. The same extension reduced production of another protein, NilC, suggesting the effect of the extension is not specific to Tdk. Long-form Tdk protein was as stable as short-form Tdk protein, and its levels were not affected by the absence of Lon or ClpP proteases. Therefore the difference in long versus short-Tdk protein levels is not likely due to differential proteolysis of the two protein forms. The long-form *tdk *mRNA transcript was present at a 1.7-fold lower level than short-form *tdk *transcript and increased levels of transcript are sufficient to overcome low protein levels, supporting the hypothesis that the low level of long-form Tdk production is due in part to low long-form *tdk *transcript levels. The bacterial N-end rule [8] states that proteins containing N-terminal arginine, lysine, leucine, phenylalanine, tyrosine and tryptophan residues are particularly sensitive to Clp-dependent degradation, reducing their *in vivo *half-lives. The phenomenon reported herein is distinct from the N-end rule as none of the known destabilizing residues occur in the MDGP N-terminal extension, nor does the extension result in protein degradation by Clp (Fig. 2D). Since the same pET-28a(+)-based expression system was used for both Tdk and NilC-trunc production, the observed phenotype may result from a combination of the MDGP extension and the pET-28a(+) expression system. These results may be relevant to researchers attempting to limit production of potentially toxic proteins from the pET-28a(+) expression plasmid under non-inducing conditions in an *E. coli *host, while still allowing protein overproduction under inducing conditions.

## Methods

### Bacterial strains

Strain KY895 ([9]; *λ*^- ^*tdk*-1 IN*(rrnD-rrnE)*1, *ilv*-276) was obtained from the *E. coli *Genetic Stock Center, strain BL21 Star™ (DE3) (F^-^*ompT hsdS*_*B *_(r_B_^- ^m_B_^-^) *gal dcm rne1*31 (DE3)) was obtained from Invitrogen (Carlsbad, CA) and strain BL21 (DE3) (F^- ^*ompT hsdS*_*B *_(r_B_^- ^m_B_^-^) *gal dcm *(DE3)) was obtained from Novagen (Madison, WI). To construct the *E. coli *KY895 *clpP *mutant strain (HGB871), primers clpPtetKWfor and clpPtetKWrev (Table 1) were used to amplify the tetracycline resistance gene from Tn*10*. The primers have 40 nt of *clpP *sequence at their 5' ends. The amplified fragment was electroporated into *E. coli *DY330 (W3110 Δ*lacU169 gal490 λc1857 *Δ(*cro-bioA*)) as described elsewhere [10]. Under the conditions used, this strain allowed homologous recombination of the linear PCR product into the DY330 chromosomal *clpP *locus, replacing the region beginning 2 bp 5' of the *clpP *start codon and ending 2 bp 3' of the *clpP *stop codon with a tetracycline resistance marker. P1 transduction was used to move the mutated locus into *E. coli *strain KY895 using standard techniques and selecting for desired transductants on tetracycline. PCR analysis was used to confirm the insertion.

**Table 1:** Oligonucleotide primers used in this study^a^

**Oligonucleotide name**	**Sequence (5'->3')^b^**	**Target ^c^**
BamHInilC	GGATCCGACGATGCCTTAATGCGACAG	*nilC*
BamHItdk	GGATCCCTTAGTGATTTTATACG	3' of *tdk*
*clpP*tetKWfor^d^	*ATCGGTACAGCAGGTTTTTTCAATTTTATCCAGGAGACGG* CAAGAGGGTCATTATATTTCG	5' of *E. coli clpP*/Tn*10 *(tet^r^)
*clpP*tetKWrev^d^	*GCCGCCCTGGATAAGTATAGCGGCACAGTTGCGCCTCTGG* GACTCGACATCTTGGTTACCG	3' of *E. coli clpP/ *Tn*10 *(tet^r^)
Ecrecaminfor	GAAAGCGGAAATCGAAGGCG	*E. coli recA*
Ecrecaminrev	CATCACACCAATTTTCATACGG	*E. coli recA*
EctdkQPfor	TTTGGTGCCGGGAAAGTC	*E. coli tdk*
EctdkQPrev	CTTGTTGTCTGGTTAAAAACTGG	*E. coli tdk*
NcoIMAGPNilC	CCATGGCCGGGCCAGCTAGAGGAGGGGGTTCTCACC	*nilC*
NcoIMDAPNilC	CCATGGACGCGCCAGCTAGAGGAGGGGGTTCTCACC	*nilC*
NcoIMDGANilC	CCATGGACGGGGCAGCTAGAGGAGGGGGTTCTCACC	*nilC*
NcoIMDGPNilC	CCATGGACGGGCCAGCTAGAGGAGGGGGTTCTCACC	*nilC*
NcoINilC	CCATGGCTAGAGGAGGGGGTTCTCACC	*nilC*
tdkprext2	AATAAAAATAAAGCTGAGCC	*tdk*
XntdkQPfor2	CTATCCGCTGATGCTTTGTTG	*tdk*
XntdkQPrev2	TACAATTTCACAAAGCTGCTC	*tdk*

^a^Additional oligonucleotides used in this study are listed in Table 2

^b^Underlined nucleotides indicate engineered restriction sites used in cloning

^c^Oligonucleotide primers are specific for *X. nematophila *unless noted otherwise; Integrated DNA Technologies (Coralville, IA) synthesized the oligonucleotides used in this study.

^d^Italicized nucleotides indicate *clpP*-region sequence

### Growth conditions

Cultures were grown in a tube roller at 30°C in Luria-Bertani (LB) broth [11] except for the FUdR growth assays, which were performed in semi-defined medium containing 5-fluorodeoxyuridine ([12]; FUdR obtained from Fisher Scientific, Pittsburgh, PA) at 37°C with shaking in a microplate reader (Molecular Devices, Sunnyvale, CA) as described previously [3]. LB agar (20 g l^-1^) plates and all liquid media were supplemented when appropriate with kanamycin (kan; 20 μg ml^-1^), chloramphenicol (cam; 20 μg ml^-1^), isopropyl-β-D-thiogalactopyranoside (IPTG; 0.2 mM) or arabinose (0.2%). M9 medium was prepared as described elsewhere [13]. Permanent stocks of cultures were stored at -80°C in dark-stored LB broth supplemented with 10% dimethylsulfoxide.

### Production of recombinant *X. nematophila *Tdk forms in *E. coli*

DNA manipulations and transformation of *E. coli *were performed using standard protocols ([14] and product literature). To construct plasmids expressing various forms of *X. nematophila *ATCC19061 *tdk *[GenBank:AY363171] [15], the BamHItdk primer (Table 1) was combined with each primer listed in Table 2 in a PCR using either *X. nematophila *(HGB007; laboratory stock of ATCC19061) chromosomal DNA or a cloned copy of *X. nematophila tdk *as template DNA. Where a proline residue was desired ahead of other tested residues, an extra alanine codon was engineered between the methionine and proline codons to allow the NcoI restriction site (which includes the translational start site) to be used for cloning. The amplified products from each primer set were cloned into plasmid pET-28a(+) (Novagen, Madison, WI) using the engineered NcoI and BamHI sites. The resulting plasmids, listed in Table 2, in addition to plasmid pTara (arabinose-inducible expression of the T7 RNA polymerase; [16]), were transformed into *E. coli *KY895, BL21 Star™ (DE3), KY895 *clpP *and BL21 (DE3). As a control, pET-28a(+) with no insert was transformed with plasmid pTara into these strain backgrounds.

### Production of truncated *X. nematophila *NilC (NilC-trunc) forms in *E. coli*

A portion of *X. nematophila nilC *was PCR-amplified from *X. nematophila *genomic DNA using primers NcoINilC or NcoIMDGPNilC and BamHINilC (Table 1), resulting in *nilC *production starting 3', at codon 22, of its predicted signal sequence-encoding region and with an additional 5' alanine codon (GCU). NcoIMDGPNilC encodes an additional four codons, for the amino acid series MDGP, before the added alanine codon, to create long-form NilC. Primers NcoIMAGPNilC, NcoIMDAPNilC and NcoIMDGANilC (Table 1) were used to encode the additional amino acid series MAGP, MDAP, and MDGA, respectively, before the added alanine codon. Products from the amplification reactions were cloned into pET-28a(+) as described above. The resulting plasmids, pETnilC, pETMDGPNilC, pETMAGPNilC, pETMDAPNilC and pETMDGANilC, were transformed with pTara into KY895 for protein production analysis.

### Immunoblot detection of Tdk and NilC

For immunoblot detections, samples from overnight cultures of KY895 carrying pTara and the appropriate pET-28a(+)-derived vector were electrophoresed and transferred to 0.2 μm PVDF membrane (Bio-Rad) using standard protocols. Immunoblots were performed using the ECL Plus Western Blotting Kit (Amersham Biosciences, Piscataway, NJ) and a goat anti-rabbit IgG horse radish peroxidase-conjugated secondary antibody (Pierce, Rockford, IL). Anti-Tdk serum was obtained from a bleed of a New Zealand White rabbit at the University of Wisconsin Laboratory Animal Resources polyclonal antibody facility following a series of injections of purified 6× his-tagged *X. nematophila *Tdk [17]. Anti-Tdk and anti-NilC sera [5] were used at a final dilution of 1:5,000. Fluorescence was detected on a Storm860 Phosphorimager (Amersham Biosciences, Piscataway, NJ).

### Primer extension mapping of short-and long-form transcripts

Total-cell RNA was isolated from KY895 carrying plasmids pTara and pETSTdk or pETLTdk following overnight growth in LB/kan/cam broth to OD_600 _= 2.0. A PAGE-purified primer, tdkprext2 (Table 1), was end-labeled using T4 Polynucleotide Kinase and [γ^32^-P]ATP (Perkin-Elmer Biosciences, Wellesley, MA) for 10 min at 37°C and the labeled primer used in cycle sequencing reactions with components of the *fmol*^® ^DNA Cycle Sequencing System kit (Promega, Madison, WI) and either plasmid pETSTdk or pETLTdk, as indicated in Figure 3. Labeled primer was also hybridized to 5 μg of total-cell RNA by dissociation at 80°C for 10 min, followed by a slow cooling to 37°C. The primer was extended by avian myeloblastosis virus reverse transcriptase enzyme (AMV-RT, Promega, Madison, WI) at 37°C and the extension products and completed sequencing reactions were resolved on a 12% SDS-polyacrylamide gel containing 8 M urea. The resulting gel was dried and visualized by exposure to a phosphor screen overnight, which was scanned on a Storm 860 phoshorimager (Amersham Biosciences, Piscataway, NJ), and the data analyzed with ImageQuant software (Amersham Biosciences, Piscataway, NJ).

### Quantitative PCR to measure relative transcript levels

*E. coli *KY895 carrying plasmids pTara and either pET-28a(+), pETSTdk or pETLTdk was subcultured from an overnight culture and grown to OD_600_~0.85 in LB broth with 0.2% glucose. Two independent cultures started from individual colonies were used for each strain. Total RNA was isolated using TRIzol (Invitrogen, Carlsbad, CA) and the RNA was DNase treated and used to make cDNA with random hexamer primers (Integrated DNA Technologies, Coralville, IA) and AMV-RT. As a control to detect DNA contamination of the DNased RNA, samples with no added AMV-RT were analyzed by PCR for amplification of *E. coli tdk *using primers EctdkQPfor and EctdkQPrev (Table 1) and, as expected, none showed product. Reactions for real-time PCR were performed in duplicate in a total volume of 25 μl with iQ™ SYBR^® ^Green Supermix (Bio-Rad, Hercules, CA), cDNA template, appropriate primers and a three-step cycling protocol on a Bio-Rad iCycler and analyzed with Bio-Rad iCycler iQ™ software. The amount of *X. nematophila tdk *transcript was measured by amplification with XntdkQPfor2 and XntdkQPrev2 primers (Table 1). As a negative control, water was used in place of cDNA template. Cycle threshold results for each sample were adjusted according to *E. coli recA *levels (amplified with Ecrecaminfor and Ecrecaminrev primers, Table 1, designed from published *E. coli recA *sequence) and then converted to arbitrary units factoring in a two-fold change in PCR product per cycle.

### Pulse labeling and immunoprecipitation

Cells were grown to OD_600 _= 0.3 in LB/kan/cam, washed and resuspended in M9 containing 0.4% arabinose, 1 μg ml^-1 ^thiamine, kan and cam and incubated for 1 h before being pulse labeled with 20 mCi/ml [^35^S]-L-methionine (PerkinElmer Life Sciences, Boston, MA) for 3 min at 37°C. Unlabeled L-methionine was added to 300 μM and 50 μl samples were removed and added to 50 μl of a 125 mM Tris-Cl, 4% SDS, pH 6.8 buffer at 20 s, 2 min and 5 min after addition of unlabeled methionine. After immediate freezing in dry ice/ethanol, samples were boiled for 4 min and to each was added 1 ml of immunoprecipitation buffer (50 mM Tris-Cl, 150 mM NaCl, 5 mM EDTA, 1% Triton X-100, pH7.5). The samples were centrifuged and supernatants incubated with 2 μl rabbit anti-Tdk serum with rocking for 1 h at 4°C. The samples were then incubated with Protein A immobilized on sepharose CL-4B (Sigma, St. Louis, MO) for 1 h at 4°C, with rocking. The pelleted beads were washed 3 times in immunoprecipitation buffer containing 0.1% SDS, separated on a 12% acrylamide denaturing gel and the gel was dried and exposed to a phosphor screen and analyzed as for the primer extension reactions.

## Authors' contributions

SSO performed the experiments and participated in experimental design and manuscript drafting and editing. HGB participated in experimental design and manuscript drafting and editing. Both authors read and approved the final manuscript.
